# Supplementary material for: Establishment and Comparison of Pathogenicity and Related Neurotropism in Two Age Groups of Immune Competent Mice, C57BL/6J Using an Indian Isolate of Chikungunya Virus (CHIKV)
Source: Viruses. 2019 Jun 25;11(6):578. doi: 10.3390/v11060578 (PMC6631960; doi:10.3390/v11060578)
Supplement: Supplementary file 1 [file viruses-11-00578-s001.pdf]

Suppl. Table 1: Evaluation of 15 CHIKV isolates for pathogenesis in 8 weeks old mice.

	8 weeks old mice														
	Day 3			Day 6			Day 9			Day 12			Day 15		
Strain n Name	Morbidity (scale of 1-10)	Limb thickness (fold change)	Survivability (%)	Morbidity (scale of 1-10)	Limb thickness (fold change)	Survivability (%)	Morbidity (scale of 1-10)	Limb thickness (fold change)	Survivability (%)	Morbidity (scale of 1-10)	Limb thickness (fold change)	Survivability (%)	Morbidity (scale of 1-10)	Limb thickness (fold change)	Survivability (%)
**CHIKV #01**	4	0.5×	100	5.5	2×	100	6.25	3×	100	9	4×	66.66	10	3.5×	16.66
**CHIKV #02**	0	No change	100	2	No change	100	3	No change	100	4	0.5×	100	4	0.2×	100
**CHIKV #06**	2	2×	100	Dead											
**CHIKV #14**	0	No change	100	2	No change	100	3	0.5×	100	4	0.2×	100	4	0.25×	100
**CHIKV #15**	0	No change	100	0	No change	100	2	0.5×	100	2	0.1×	100	3.56	0.1×	100
**CHIKV #07**	0	No change	100	2	No change	100	3	0.5×	100	4	0.2×	100	4.25	0.2×	100
**CHIKV #10**	0	No change	100	3	No change	100	3	No change	100	4	No change	100	5	No change	100
**CHIKV #32**	0	No change	100	0	No change	100	0	No change	100	1	No change	100	2	No change	100
**CHIKV #40**	0	No change	100	0	No change	100	0	No change	100	1	No change	100	2	No change	100
**CHIKV #42**	0	No change	100	0	No change	100	0	No change	100	0	No change	100	2	No change	100
**CHIKV #11**	3	No change	100	4.5	0.75×	100	5.56	1.25×	100	7	3×	100	7	2.5×	83.33
**CHIKV #12**	2	No change	100	3.5	1.5×	100	4.25	3×	100	6.5	3×	100	6.5	2×	83.33
**CHIKV #20**	2	No change	100	4	0.8×	100	4	1×	100	5	0.12×	100	5	0.1×	100
**CHIKV #88**	0	No change	100	0	No change	100	0	No change	100	0	No change	100	0	No change	100
**CHIKV #112**	0	No change	100	0	No change	100	0	No change	100	0	No change	100	2	No change	100

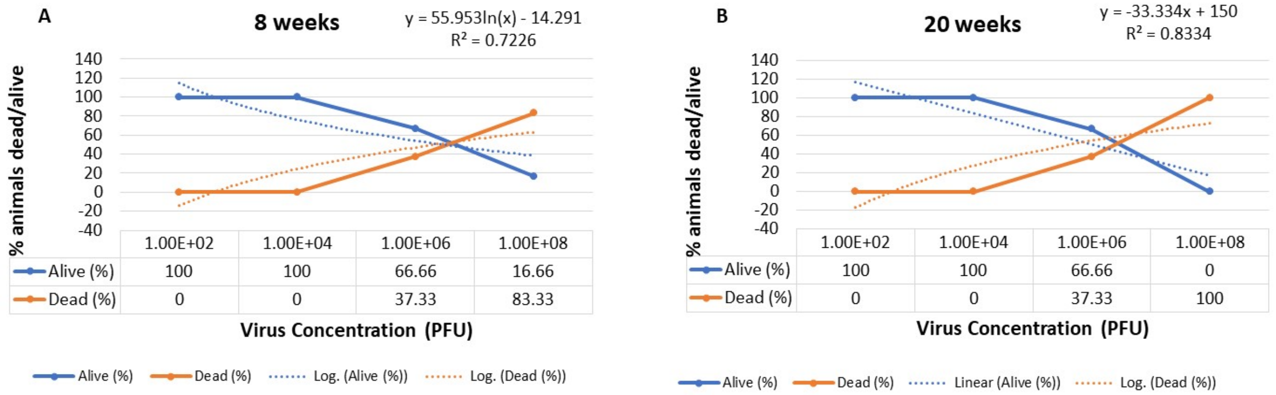

Suppl. Fig. 1: Determination of MID_{50} for CHIKV#1 in 8 weeks and 20 weeks old infected mice. Each group of the study had eight animals (four male and four female) and the animals were observed for a period of 15 days and the percentage of animals dead/alive was calculated at 15 dpi before the termination of the study

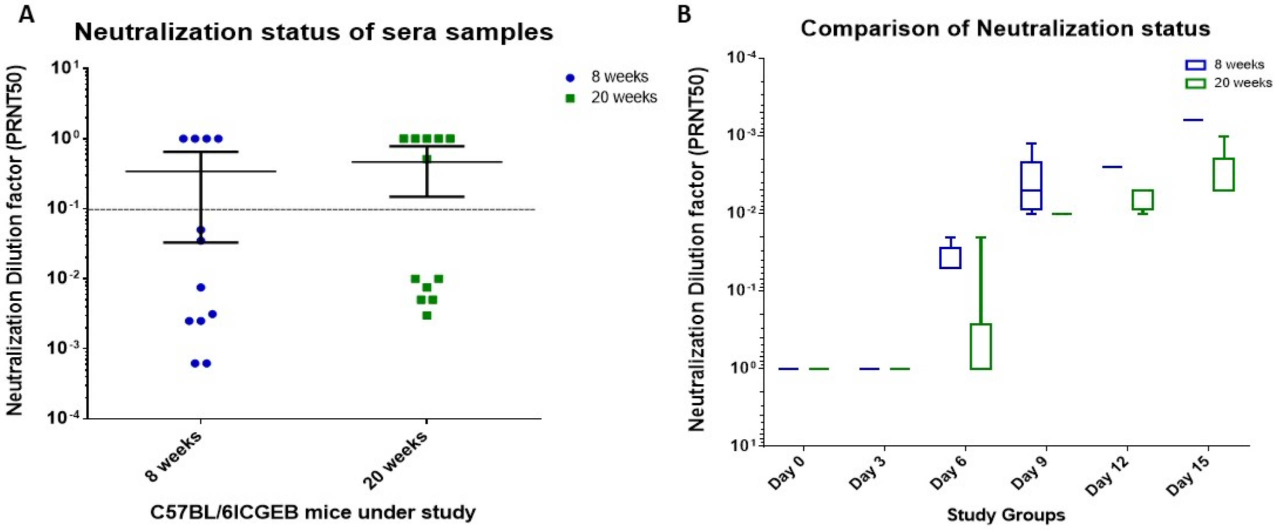

Suppl. Fig 2: Comparison of the neutralization status of the binding antibodies in 8 week vs. 20 weeks old mice (2a and 2b)

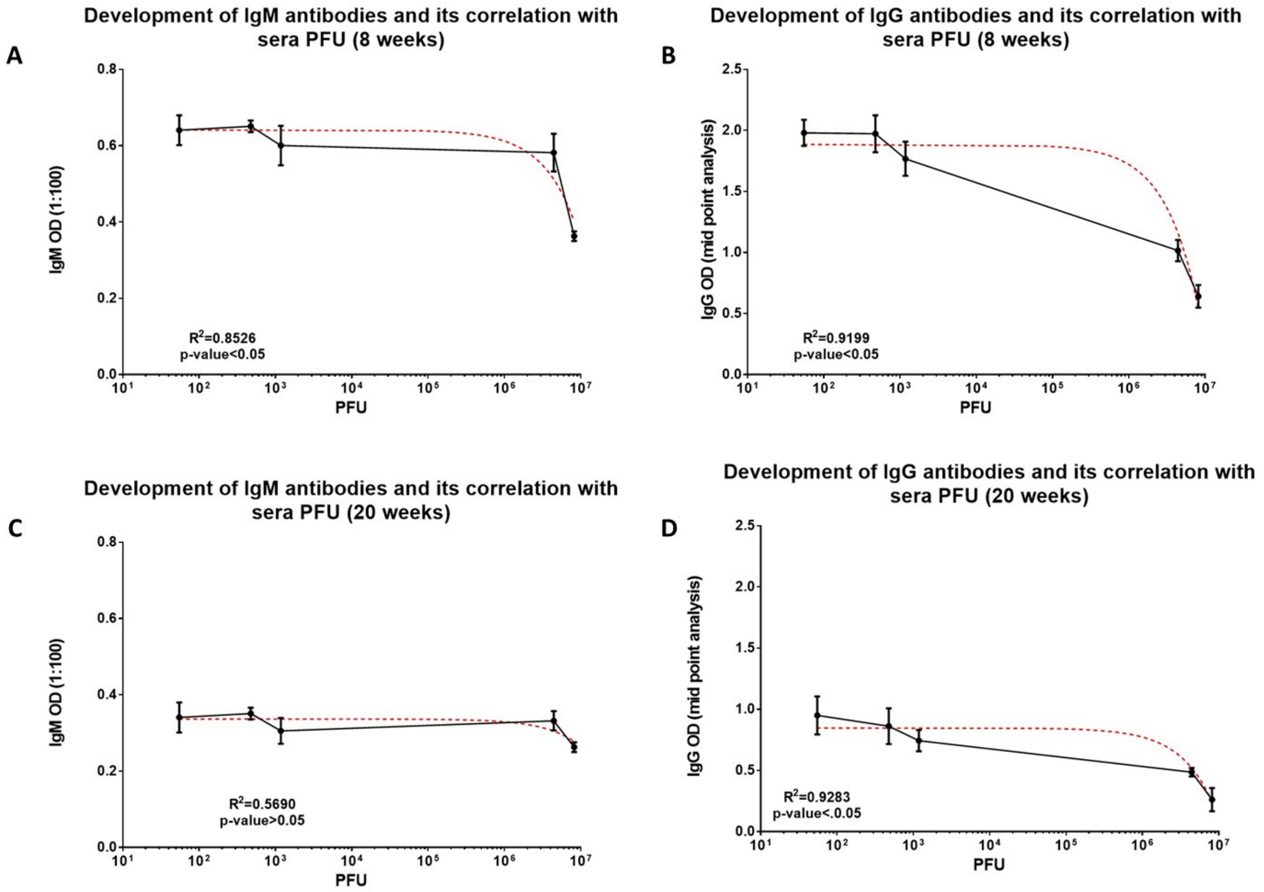

Suppl. Fig 3: Correlation of binding Abs and viral load in 8 weeks (3a and 3b) and 20 weeks old infected mice (3c and 3d).
